# Data-based decision-making at all managerial levels in health care: an integral part of evidence-based practice

**DOI:** 10.1186/1472-6963-14-S2-P40

**Published:** 2014-07-07

**Authors:** Leon Epstein

**Affiliations:** 1 School of Public Health and Community Medicine, Jerusalem, Israel; 2 lsrael Academic College, Ramat Gan, Israel

## 

While the critical role of an evidence base for policy decision-making in health care is obvious and well documented, the same cannot be said for daily decision making by health care managers at all levels of the system.

The relevance for heads of clinical departments, chief nurses, and specialist units became clear when directing a University Hospital and later being part of the management of a similar institution some years later. To be clear, this is not related to clinical decisions but to those in the managerial role. Two issues were immediately clear - there was the inherent principle of the “I know” method of decision making -”don’t confuse me with facts”, and secondly that most heads of service departments had not received training in decision-making. It was taken for granted that that they knew how to do it. An additional issue was the lack of relevant information on issues that required a manager’s decision. There was a need to plan studies, completed in relatively short time periods, in order to allow for “real-time” decision making.

On the basis of the above analysis, a course was designed for students within the framework of studies for the Master of Public Health or Master of Health Administration. The syllabus includes both models for administrative decision- making (adapted to health care) as well as a series of case studies that had been performed over the years and provided a practical basis for the managerial decision-making process. These included, amongst others:

• The performance of unnecessary laboratory tests in the community - reasons for ordering, use of the results, and effect of intervention

• The implication of low reliability of interpretation of radiological tests and their possible improvement

• Inappropriate use of hospitalization days

• The use of routine computerized data for quality assessment in hospitals

All students were required to present assignments on defined issues.

This included epidemiological data, need for additional manpower and its training and financial implications. The defined tasks included:

• The need for additional manpower

• The introduction of a new test or a change in method of performance

• Rise in infant mortality in a defined region

In addition a final project required the preparation of a detailed plan relating to a question requiring a decision in a health service.

The course has been very positively evaluated over the years especially in having provided a defined approach to health managerial decision making.

